# Direct detection of bacteremia by exploiting host-pathogen interactions of lipoteichoic acid and lipopolysaccharide

**DOI:** 10.1038/s41598-019-42502-5

**Published:** 2019-04-17

**Authors:** Jessica Z. Kubicek-Sutherland, Dung M. Vu, Aneesa Noormohamed, Heather M. Mendez, Loreen R. Stromberg, Christine A. Pedersen, Astrid C. Hengartner, Katja E. Klosterman, Haley A. Bridgewater, Vincent Otieno, Qiuying Cheng, Samuel B. Anyona, Collins Ouma, Evans Raballah, Douglas J. Perkins, Benjamin H. McMahon, Harshini Mukundan

**Affiliations:** 1 Physical Chemistry and Applied Spectroscopy, Chemistry Division, Los Alamos National Laboratory, Los Alamos, New Mexico United States; 2 Department of Biomedical Engineering, University of New Mexico, Albuquerque, New Mexico United States; 3 University of New Mexico/KEMRI Laboratories of Parasitic and Viral Diseases, Centre for Global Health Research, Kenya Medical Research Institute, Kisumu, Kenya; 4 Center for Global Health, Department of Internal Medicine, University of New Mexico Health Sciences Center, Albuquerque, New Mexico United States; 5 Department of Medical Biochemistry, School of Medicine, Maseno University, Maseno, Kenya and University of New Mexico/KEMRI Laboratories of Parasitic and Viral Diseases, Centre for Global Health Research, Kenya Medical Research Institute, Kisumu, Kenya; 6 Department of Biomedical Sciences and Technology, School of Public Health and Community Development, Maseno University, Maseno, Kenya and University of New Mexico/KEMRI Laboratories of Parasitic and Viral Diseases, Centre for Global Health Research, Kenya Medical Research Institute, Kisumu, Kenya; 7 Department of Medical Laboratory Science, School of Public Health, Biomedical Sciences and Technology, Masinde Muliro University of Science and Technology, Kakamega, Kenya and University of New Mexico/KEMRI Laboratories of Parasitic and Viral Diseases, Centre for Global Health Research, Kenya Medical Research Institute, Kisumu, Kenya; 8 Theoretical Biology and Biophysics, Theoretical Division, Los Alamos National Laboratory, Los Alamos, New Mexico United States

**Keywords:** Microbiology, Applied microbiology

## Abstract

Bacteremia is a leading cause of death in sub-Saharan Africa where childhood mortality rates are the highest in the world. The early diagnosis of bacteremia and initiation of treatment saves lives, especially in high-disease burden areas. However, diagnosing bacteremia is challenging for clinicians, especially in children presenting with co-infections such as malaria and HIV. There is an urgent need for a rapid method for detecting bacteremia in pediatric patients with co-morbidities to inform treatment. In this manuscript, we have developed and clinically validated a novel method for the direct detection of amphiphilic pathogen biomarkers indicative of bacteremia, directly in aqueous blood, by mimicking innate immune recognition. Specifically, we have exploited the interaction of amphiphilic pathogen biomarkers such as lipopolysaccharides (LPS) from Gram-negative bacteria and lipoteichoic acids (LTA) from Gram-positive bacteria with host lipoprotein carriers in blood, in order to develop two tailored assays – lipoprotein capture and membrane insertion – for their direct detection. Our assays demonstrate a sensitivity of detection of 4 ng/mL for LPS and 2 ng/mL for LTA using a waveguide-based optical biosensor platform that was developed at LANL. In this manuscript, we also demonstrate the application of these methods for the detection of LPS in serum from pediatric patients with invasive *Salmonella* Typhimurium bacteremia (n = 7) and those with *Staphylococcal* bacteremia (n = 7) with 100% correlation with confirmatory culture. Taken together, these results demonstrate the significance of biochemistry in both our understanding of host-pathogen biology, and development of assay methodology, as well as demonstrate a potential new approach for the rapid, sensitive and accurate diagnosis of bacteremia at the point of need.

## Introduction

Bacteremia, the presence of bacteria in the bloodstream, can be either asymptomatic or associated with actively multiplying organisms causing life-threatening infection or sepsis^1^. Sepsis is the most common cause of global childhood mortality^2–5^, which is influenced by several factors including, but not limited to, the nature of the pathogen, the presence of co-morbidities (HIV/AIDS, malaria), and the timeliness of therapeutic intervention^3–5^. Thus, children from high-disease burden regions, with high incidence of co-morbidities, are at the highest risk of death caused by sepsis. In a study conducted in Kilifi, Kenya, Berkeley *et al*. found that 12.8% of infants (<60 days of age) and 5.9% of children (<5 years) had bacteremia, accounting for ~26% of all in-hospital deaths^3^. Berkeley *et al*. also found 33.4% of the mortality from bacteremia occurred on the day of hospital admission, and 70.5% within two days. Similarly, in a holoendemic region of malaria transmission where the patient samples for the current study were collected (Siaya, Kenya), we found that 11.7% of the children (<5 years) presenting at hospital had bacteremia which resulted in an 8.5-fold higher mortality^4^. Both of these patient populations have all the aforementioned risk factors: endemic malaria, high incidence of HIV/AIDS, and extensive malnutrition.

These findings indicate that timely therapeutic intervention is critical for saving lives, especially in immuno-compromised individuals in high-disease burden areas^6,7^. However, available methods for sepsis diagnosis are unable to provide reliable and accurate information to guide timely treatment. The current WHO guidelines for diagnosis of bacteremia fails to identify the condition in over 1/3^rd^ of African children^8^. The diagnosis of bacteremia/sepsis is challenging for many reasons. Sepsis is not a disease, but rather a syndrome caused by a variety of Gram-positive and Gram-negative bacterial pathogens. Among Gram-negatives, invasive non-Typhi *Salmonella* (iNTS) serovars are responsible for high rates of sepsis in children under the age of 5^9^. 1,800–9,000 in 100,000 children with HIV infection in Africa are estimated to have iNTS associated bacteremia^10^. Among Gram-positives, *Streptococcus pneumoniae* and *Staphylococcus aureus* are largely implicated in pediatric sepsis^5^. Any diagnostic strategy for bacteremia/sepsis should target a suite of potential pathogens.

Microbial culture is the current gold standard for sepsis diagnosis, which is both slow and insensitive^11^. Most laboratories in developing nations are unable to perform a timely and precise diagnosis using culture^12,13^. Further, Gram-positive bacteremia is severely under-represented by culture, unless clinical samples other than blood (e.g., bone marrow) are considered^14^. More recently, several multiplexed polymerase chain reaction (PCR) methods have been described^15^. However, the rapid emergence of pathogens such as iNTS serovars sequence type 313 and emerging antimicrobial resistance challenge the reliability of this approach^16,17^. Further, during bacteremia, the circulating concentration of the pathogen in blood is very low (1 CFU/mL for iNTS)^8^. Therefore, most molecular diagnostic methods still require blood-culture for concentration of the pathogen, delaying treatment.

Sepsis results in an extensive inflammatory response in the host, which has encouraged the development of host-biomarker based diagnostics for the syndrome. Gilchrist *et al*. have reported cytokine signaling as a mechanism to predict outcomes in children with bacteremia in Malawi^18^, but the applicability of this method in multi-infection scenarios is questionable. Generic disease indicators like C-reactive protein are non-specific and offer poor prognostic value in such populations^19,20^. Thus, there remains a need for a rapid and accurate diagnostic assay that can direct therapeutic intervention at the point of need. The work presented in this manuscript is the first step towards developing such a capability and is inspired by the ability of the innate immune response to sensitively detect bacteremia in blood very early in the course of infection.

## Results

### Amphiphile immunoassay detection strategies

The innate immune system is capable of rapidly identifying all pathogens by recognizing conserved virulence factors known as pathogen associated molecular patterns (PAMPs)^21^ via pattern recognition receptors, such as the Toll-like receptors (TLRs)^22^. Many bacterial PAMPs are amphiphilic molecules, containing both hydrophobic and hydrophilic moieties. Herein, we present tailored ultra-sensitive assays for the direct detection of a suite of amphiphilic bacterial PAMPs– lipopolysaccharide (LPS) for Gram-negative pathogens and lipoteichoic acid (LTA) for Gram-positive pathogens– in blood. Activation of the immune system by LPS occurs through TLR4, whereas LTA activates the TLR2/TLR6 heterodimer^21^. Both interactions result in cytokine signaling, which is one of the primary host response features targeted for host-based diagnostic development. However, host indicators of disease provide vague therapeutic information as compared to direct detection of the PAMPs themselves. Yet, this avenue has not been extensively pursued because of the challenges associated with detecting amphiphilic bacterial PAMPs in blood.

**Figure 1:**
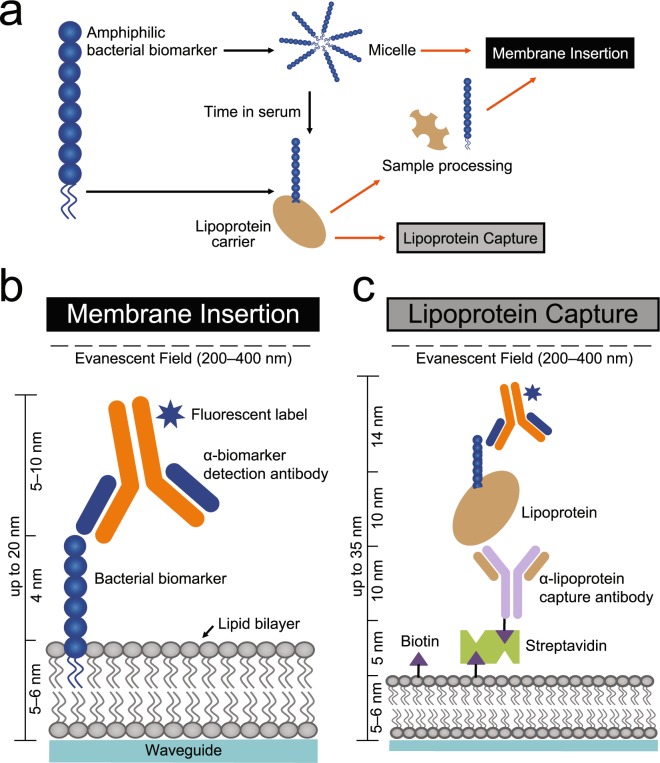
Overview of bacterial PAMP detection strategies. **(a**) Schematic representation of amphiphilic bacterial biomarkers in association with biochemically similar molecules either by forming micelles or interacting with host lipoprotein carriers. Black arrows indicate physiological associations, while orange arrows indicate experimental processes. In the absence of sequestration by a host lipoprotein carrier, bacterial PAMPs can be detected by (**b**) membrane insertion, which requires only one antibody. When associated with a host lipoprotein carrier, detection can be performed using (**c**) lipoprotein capture, which requires two antibodies, as well as prior knowledge of PAMP-lipoprotein carrier associations. Dimensions of the various layers are included in order to present the biophysical dimensions of assay performance within the evanescent field of the waveguide. Graphic representations are not drawn to scale.

LPS and LTA are both amphiphilic molecules anchored in the cell membrane by a lipid moiety with an exposed hydrophilic portion^23,24^ (Fig. 1a). We and others have found that this biochemical property makes these PAMPs unstable in aqueous blood and facilitates their interaction with host carriers, including serum binding proteins such as high- and low- density lipoproteins (HDL and LDL)^25,26^ as well as LPS binding protein (LBP)^27^. HDL and LDL are composed of a core nanodisc lipidated structure that associates with the lipid moieties of LPS^28,29^, LTA^25^ and lipoarabinomannan (LAM) from *Mycobacterium tuberculosis*^30^. However, the interaction of LPS and LTA with host lipoproteins interferes with traditional methods for detecting these bacterial biomarkers directly in patient blood^31^.

Immunoassays designed to target proteins display limited sensitivity when applied to amphiphiles in aqueous blood^31^ due to the aforementioned host-pathogen interactions that sequester the biomarker^29,30,32,33^. Herein, we evaluate the feasibility of using two novel assay methodologies that account for the amphiphilicity of LPS and LTA to achieve their ultra-sensitive detection in serum. The first assay, termed **membrane insertion** (Fig. 1b), utilizes the passive interaction between an amphiphilic biomarker and a lipid bilayer to capture the biomarker directly on the biosensor surface for interrogation with a dye-conjugated antibody. This rapid approach requiring only a single specific antibody has been previously validated for the detection of LPS^34^ from *E. coli* and LAM from *M. tuberculosis*^32,35^. The second assay, termed **lipoprotein capture** (Fig. 1c) exploits the interaction between amphiphilic biomarkers and host carrier lipoproteins in blood and thus requires prior knowledge of PAMP-lipoprotein association. In this approach, an antibody targeting the lipoprotein carrier is used to capture a host-biomarker complex to the biosensor surface followed by interrogation with a dye-conjugated antibody targeting the biomarker of interest. Lipoprotein capture has been validated for the detection of LAM from *M. tuberculosis* in patient serum^30^.

The work presented here integrates the enhanced sensitivity of a waveguide-based optical biosensor^36,37^ with assay methods that are tailored for detection of amphiphilic biomarkers. The waveguide-based optical biosensor platform utilizes single mode planar optical waveguides to achieve ultra-sensitive detection within the evanescent field of the waveguides. Light from the laser is coupled into gratings etched on the waveguide, generating an evanescent field (200–400 nm from the surface) within which biodetection is accomplished. This allows for the separation of surface bound components from contaminants in solution, greatly reducing non-specific interactions and enhancing sensitivity of detection. This, combined with the binding affinity of the antibodies used in the assay contributes to the sensitivity of the assays, which are consistently at least an order of magnitude higher than that achieved in conventional immunoassay platforms.

### Detection of LPS in human serum

Detection of bacterial PAMPs directly in human blood serum, the component of blood that does not contain blood cells or clotting factors, was performed on a waveguide-based biosensor developed at LANL^36,38,39^. The biosensor performs ultra-sensitive measurements using waveguides by probing only surface-bound PAMPs captured *via* one of our immunoassay strategies (Fig. 1b,c). Detection is performed within an evanescent field extending 200–400 nm from the surface to minimize the indirect detection of contaminants in solution. Characterization of the waveguide surface and each of the layers therein has been previously reported by us, and other investigators^32,36,37,39,40^. The waveguide is functionalized with a supported DOPC bilayer, which is about 5–6 nm in height^41,42^. Native HDL is a discoidal bilayer stabilized by two molecules of APOA1. Sligar *et. al*. have modeled the dimensions and behavior of HDL nanodiscs and demonstrate a 10 nm size of native HDL nanodiscs^42,43^, such as the ones used in this study. Antibodies measure 5–15 nm when measured by atomic force microscopy^44^. The exact orientation of LPS or LTA on the supported lipid bilayer architecture is unclear. Even with the associated antigen (whose dynamics can vary depending on the position, but never more than 4 nm)^38,45^, the immobilized layer is thus within the confines of the evanescent field in order to facilitate detection using the waveguide-based biosensor (Fig. 1b,c). Measurements are extremely rapid (3 seconds), yield minimal background signals, and can be adapted to the simultaneous detection of a variety of fluorescent labels. We have previously validated the application of this technology for the detection of anthrax^46,47^, influenza^48^, breast cancer^41^ and tuberculosis^49–51^.

**Figure 2:**
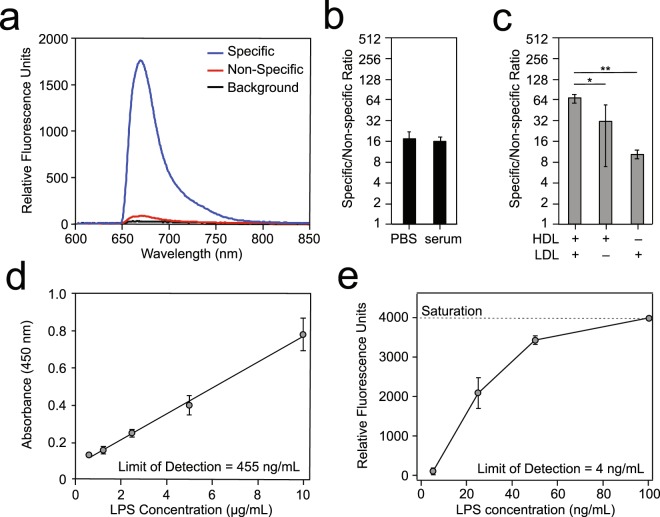
Assay optimization for the detection of LPS in human serum. (**a)** Representative measurement of LPS (25 ng/mL) incubated overnight at 4 °C in control human serum, with the specific signal (RFU) from the detection α-LPS antibody (25 nM) as a function of emission wavelength (nm). The background and non-specific signals are measured before the addition of LPS. **(b)** Detection using membrane insertion of 50 ng/mL of LPS derived from *S*. Typhimurium ATCC 14028 following dilution in PBS or overnight incubation in control human serum. Data are presented as the specific/non-specific (S/N) ratio. (**c**) Detection using lipoprotein capture of 50 ng/mL of LPS derived from *S*. Typhimurium ATCC 14028 following overnight incubation in control human serum using capture antibodies targeting HDL and/or LDL. **(d)** Concentration dependent detection of *S*. Typhimurium LPS using lipoprotein capture with 25 nM of the α-LPS antibody in ELISA format measured in absorbance at 450 nm. (**e**) Concentration dependent detection of *S*. Typhimurium LPS using lipoprotein capture in the LANL waveguide-based biosensor. All values given in (**b**–**e**) are the mean  ±  standard deviation derived from at least two independent determinations (n = 2). ELISA assays were performed in triplicate (n = 3). Statistical significance was determined by one-way ANOVA with Fisher’s least significant difference test used for *post hoc* analysis (**P < 0.01 or *P < 0.05).

In this study, we utilized a 635 nm laser to excite the detection antibodies labeled with Alexa Fluor® 647 (AF647). A representative measurement for the detection of 25 ng/mL LPS spiked in human serum is shown in Fig. 2a. The antibodies chosen for this assay were evaluated for cross-reactivity. LPS and LTA are extremely conserved bacterial antigens, which is the primary reason they were chosen for the development of this platform for the diagnosis of sepsis. However, in order to determine the false negative and more importantly, the false-positive rates, the antibodies were screened thoroughly before use in the assays shown in the manuscript. The antibodies were screened against the antigen of choice, and also similar antigens from across multiple species. In addition, all LPS antibodies were screened against LTA, and vice versa, in order to provide a thorough assessment of the cross-reactivity in diagnostic application. The data for the selected antibodies are presented in Fig. S1. The α-LPS antibody only binds to *S*. Typhimurium LPS and not any of the other LPS or LTA samples. The α-Gram+ and α-Sau antibody cocktail did not bind to any LPS samples, but was able to detect all LTA samples tested.

The waveguide-associated background of 20 relative fluorescent units (RFU) is indicative of scattering by intrinsic impurities associated with the waveguide itself. The non-specific signal is the background fluorescence measurable with the labeled detection antibody, in this case the α-LPS antibody AF647 conjugate (81 RFU), in the absence of the LPS antigen. The specific signal of 1785 RFU is the signal measured after the addition of LPS. The specific/non-specific ratio (S/N) for this representative assay is 29, which was calculated as shown in Eq. (1).

Next, we compared the relative performance of the membrane insertion (Fig. 1b) and lipoprotein capture (Fig. 1c, Table S1) assays for direct detection of LPS in patient serum. Prior to measurements, LPS was either incubated in PBS (no lipoproteins) or overnight at 4 °C in control human serum to allow time for the dynamic interactions between serum components and LPS to occur. When 50 ng/mL LPS was detected by membrane insertion, we obtained a S/N ratio of 16 regardless of whether LPS was diluted in PBS or incubated in human serum (Fig. 2b, Table S5). In contrast, the LPS S/N ratio significantly increased by 4-fold when using the lipoprotein capture assay (HDL + LDL: *P* < 0.01, Fig. 2c, Table S5). This assay relies on the pull-down of HDL and LDL nanodiscs found in blood, allowing for the recovery and detection of any associated LPS molecules. We have found that while the individual capture antibodies were able to pull down the HDL (α-apoAI) and LDL (α-apoB) nanodiscs, respectively, the additive effects of using a cocktail of both these capture antibodies appear to be much more effective in capturing the LPS biomarker^29,52^. The combination of targeting HDL and LDL together shows a synergistic affect as compared to either HDL (*P* < 0.05) or LDL (*P* < 0.01) alone (Fig. 2c, Table S5). Therefore, the detection of LPS in serum is more sensitive using the lipoprotein capture assay targeting both HDL and LDL as compared to the membrane insertion assay.

While these assays were developed on a waveguide-based biosensor platform, the transduction strategy can be applied to any immunoassay format. To facilitate broader use of our assay methods and to explore sensitivity and specificity of detection, we adapted the lipoprotein capture assay for the detection of PAMPs using an enzyme-linked immunosorbent assay (ELISA) platform. Serum-coated 96-well microtiter plates were incubated with LPS to allow capture by lipoproteins, the complexes of which were interrogated with the α-LPS antibody, and a subsequent secondary-enzyme conjugated antibody. LPS was detected at varying concentrations (Fig. 2d) yielding a limit of detection of 455 ng/mL [Eq. (2)]. Conversely, detection of LPS using the lipoprotein capture assay in the waveguide-based biosensor platform displayed a limit of detection of 4 ng/mL, over 100-fold increase in sensitivity (Fig. 2e, Table S1). Similarly, we previously reported over 1000-fold increase in sensitivity for the detection of LAM from *M. tuberculosis* in serum on the biosensor platform as compared to ELISAs^51^. Thus, although the assays can be adapted to other conventional sensor platforms, the compromise in sensitivity may limit their clinical utility in some formats.

### Detection of LTA in human serum

**Figure 3:**
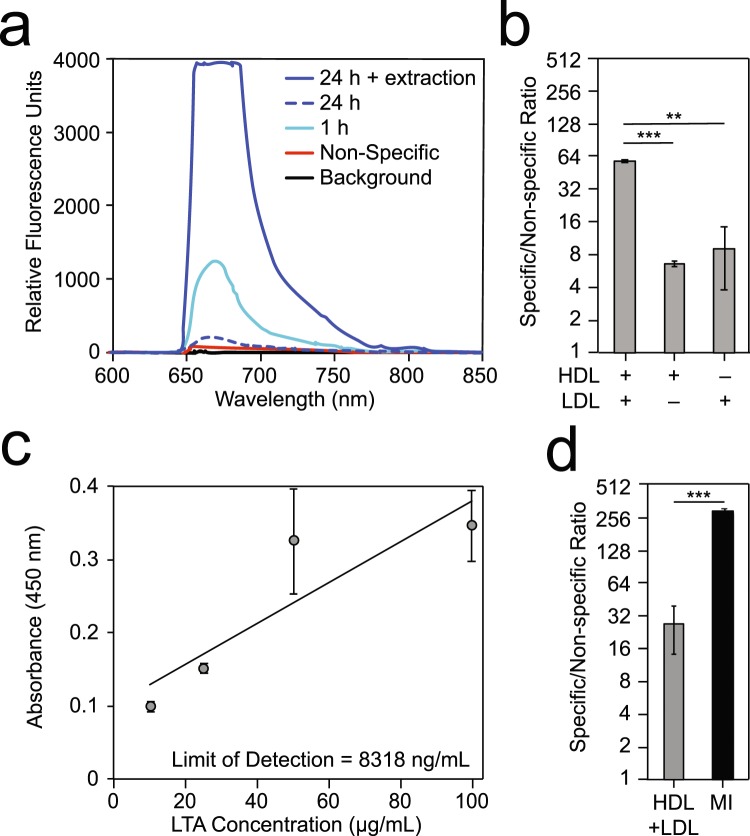
Assay optimization for the detection of LTA in human serum. (**a**) Membrane insertion assay using the 25 nM α-Gram+ antibody for the detection of *S. aureus* LTA (1 µg/mL) incubated in control human serum with 1 h incubation at room temperature (teal line), 24 h incubation overnight at 4 °C (dotted blue line), and 24 h incubation overnight at 4 °C followed by sample processing (blue line). The background and non-specific signals are measured before the addition of LTA. **(b)** Detection using lipoprotein capture with the 25 nM α-Gram+ antibody to detect 1 µg/mL of *S. aureus* LTA following overnight incubation in control human serum using capture antibodies targeting HDL and/or LDL. **(c)** Concentration dependent detection of *S. aureus* LTA using lipoprotein capture with 25 nM of the α-LTA antibody in ELISA format measured in absorbance at 450 nm. **(d)** Comparison of the sensitivity of lipoprotein capture (HDL and LDL together) and membrane insertion assays for the detection of 100 ng/mL *S. aureus* LTA using the cocktail of 25 nM α-Gram+ monoclonal and 25 nM of α-Sau polyclonal antibody. All values given in **(b–d)** are the mean  ±  standard deviation derived from at least two independent determinations (n = 2). ELISA assays were performed in triplicate (n = 3). Statistical significance was determined by one-way ANOVA with Fisher’s least significant difference test used for *post hoc* analysis (***P < 0.001 or **P < 0.01).

The membrane insertion and lipoprotein capture strategies were evaluated for the direct detection of LTA in human serum. Incubation of 1 µg/mL *S. aureus* LTA in control human serum for 24 h greatly reduced the signal observed compared to 1 h incubation by membrane insertion (Fig. 3a). We hypothesized that the formation of LTA-host lipoprotein complexes following incubation in serum was mainly responsible for the signal loss. To evaluate this, we developed a rapid strategy to liberate LTA from the host lipoproteins. This sample processing method greatly enhances the sensitivity of the membrane insertion assay by releasing LTA from serum in less than 2 minutes using a modified chloroform-methanol lipid extraction protocol (Fig. 3a, see Methods).

The comparative detection of 1 µg/mL *S. aureus* LTA in human serum using lipoprotein capture was performed by pulling down either HDL, LDL or both and then interrogating for LTA using the α-Gram+ antibody (Fig. 3b, Table S2). As seen with LPS, there is a synergistic effect in using a combination of HDL and LDL capture antibodies as compared to using either HDL (*P* < 0.001) or LDL (*P* < 0.01) alone (Fig. 3b, Table S6).

Adaptation of the lipoprotein capture assay to an ELISA plate-based format was performed using serum-coated 96-well microtiter plates incubated with LTA to allow association of LTA with serum lipoproteins. Immobilized LTA was then interrogated with the α-LTA antibody and a subsequent secondary-enzyme conjugated antibody. LTA was detected at varying concentrations (Fig. 3c) yielding a limit of detection of 8318 ng/mL [Eq. (2)].

In order to enhance the sensitivity of the LTA assays, we elected to combine the α-Gram+ monoclonal and α-Sau polyclonal antibodies in equal molar ratios and test again using the waveguide-based biosensor. Using this antibody cocktail, the detection of 100 ng/mL *S. aureus* LTA showed that the membrane insertion assay was more sensitive than the lipoprotein capture assay targeting both HDL and LDL together (*P* < 0.001, Fig. 3d) with a limit of detection of 2 ng/mL (Table S2).

### Detection of LPS and LTA directly in pediatric serum samples

Lastly, we tested the ability of our assays to detect both Gram-negative and Gram-positive bacteremia directly in 20 blinded pediatric clinical samples^4^. This study was performed as a proof-of-principle, not as a clinical trial for which larger sample numbers would be required. The samples were from pediatric patients enrolled at Siaya County Referral Hospital in western Kenya enrolled between 2004 and 2014 as part of a study on the pathogenesis of severe malarial anemia and related co-morbidities. The cohort was comprised of malaria-infected children with and without bacteremia (ages 4–25 months). The demographic information and laboratory results for the patients are displayed in Table 1.

**Table 1:** Summary of patient demographics and laboratory test results.

Patient ID^a^	Sex	Age (months)	Malaria	Biosensor	Blood Culture ID^b^	Sequence Confirmation^c^ (NCBI Accession No.)
1	F	25	+	+	*Salmonella* Gp D	*S*. Entertitidis (NHTP00000000)
2	F	21	+	+	*Salmonella* Gp B	*S*. Typhmurium ST313 (NHRB00000000)
3	F	6	+	+	*Salmonella* Typhimurium	−
4	F	15	+	+	*Salmonella* Typhimurium	−
5	F	10	+	+	*Salmonella* Gp B	*S*. Typhmurium ST313 (NHQY00000000)
6	F	5	+	+	*Salmonella* spp.	*S*. Typhmurium ST313 (NHQX00000000)
7	F	14	n.d.	+	*Salmonella* spp.	−
8	M	11	−	negative	No growth	−
9	F	4	−	negative	No growth	−
10	F	8	−	negative	No growth	−
11	F	17	−	negative	No growth	−
12	M	4	−	+	*Staphylococcus epidermidis* ^b^	−
13	M	15	+	+	*Staphylococcus aureus*	*Staphylococcus aureus* (UGA21)
14	F	11	+	+	*Staphylococcus aureus*	*Staphylococcus aureus* (NWTZ00000000)
15	M	12	+	+	*Staphylococcus* spp.^b^	−
16	M	18	+	+	*Staphylococcus* spp.^b^	−
17	M	23	+	+	*Staphylococcus* spp.^b^	−
18	M	10	+	+	*Staphylococcus* spp.^b^	−
19	M	13	+	negative	No growth	−
20	M	6	+	negative	No growth	−

^a^Patient samples numbered 1–11 were tested for LPS, while patient samples numbered 12–21 were tested for LTA.

^b^Coagulase-negative *Staphylococcus* spp.

^c^Select strains were further classified by whole genome sequencing with NCBI accession numbers included if available or bacterial strain ID if not available.

n.d. data not available.

**Figure 4:**
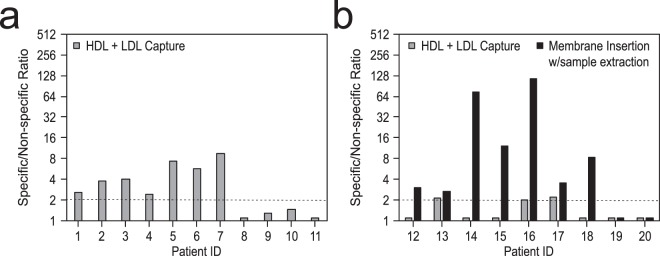
Detection of LPS and LTA directly in pediatric patient serum samples. Data are presented as the S/N ratio with a value above 2 indicating a positive result. (**a**) Detection of LPS in clinical serum samples using the lipoprotein capture assay. (**b**) Detection of LTA in clinical serum samples using both the lipoprotein capture (grey bars) and membrane insertion (black bars) assays.

The detection of LPS and LTA directly in serum (40–50 µL) was performed on the waveguide-based biosensor using the lipoprotein capture assay. The data are presented as a S/N with a value of 2 or greater indicating a positive result, which reflects the average of 20 non-specific measurements plus two standard deviations (2σ). Because of the low sample volume available from pediatric patients, samples 1–11 were tested for LPS (Fig. 4a, Table S3), while samples 12–20 were tested for LTA (grey bars, Fig. 4b, Table S4) to allow for repeated measurements. First, lipoprotein capture of HDL + LDL complexes were performed an all 20 samples. Comparison of our assay results with culture-confirmed disease status of patients with Gram-negative infection (Table 1), yielded 100% corroboration with disease status. However, in Gram-positive infected patients the lipoprotein capture assay was only able to detect 3 of 7 culture-positive samples. This is likely due to the inability of the α-Gram+ antibody to bind LTA-lipoprotein complexes at the level of sensitivity required for detection of LTA directly in the serum of bacteremia patients.

To enhance the sensitivity of the LTA assay in patient serum samples, we processed the remaining 15–20 µL of each sample (samples 12–20) using our novel method to release LTA for interrogation with the membrane insertion assay (black bars, Fig. 4b, Table S4). The sample processing procedure releases LTA from any interacting lipoproteins in the serum to allow for the direct interaction between LTA and the lipid bilayer (see Methods). Importantly, using this method, we found 100% corroboration with blood culture confirmed disease status when testing the same set of patient samples (Table 1).

## Discussion

The early diagnosis of bacteremia and initiation of antibiotic treatment is essential for mitigating the morbidity and mortality associated with sepsis^2^. Indeed, NTS is one of the major drivers of pediatric mortality in high-disease burden populations^53^. Previous work evaluating the need for point of care diagnostics for sepsis in Kenya using mathematical modeling shows that point of care diagnostics – even with poor sensitivity and specificity – can improve outcomes in high-disease burden populations^54^. Even state-of-the-art automated blood culture technologies which include BD BACTEC (BD Diagnostics), BacT/ALERT (bioMérieux) and VersaTRECK (TREK Diagnostic Systems) take hours, if not days, to detect bacteremia^55^. However, the majority of childhood sepsis deaths occur in low-income countries where malnutrition, poor sanitation, and co-morbidities increase the rate of bacteremia and the healthcare system does not support such technologies^3,4^. In contrast, the innate immune system can detect bacteremia in seconds by recognizing PAMPs^56^.

Most antibodies targeting LPS, especially polyclonally derived antibodies, demonstrate cross-reactivity to the antigen from a variety of Gram-negative bacteria. Some of them also demonstrate cross-reactivity to LTA. Such antibodies can be further examined and used for the development of a broadly sensitive assay for sepsis. However, for the purposes of this manuscript, we have chosen to work with an antibody that demonstrates minimal cross-reactivity with LPS from other species, or with LTA, so as to capture the ability of this assay to offer more targeted diagnostics as well – in this case for iNTS infections in pediatric populations. Indeed, both antibodies and LPS aptamers^57,58^, display variable reactivity with LPS derived from various Gram-negative bacteria resulting in pathogen-specific assays. Additional recognition ligands such as these can also be examined, depending on the end-application of our assay– generic detection of sepsis, or targeted detection of specific sub-groups of pathogens.

The importance of direct measurement of LPS and LTA for the diagnosis and treatment of sepsis has been noticed by several investigators, who have developed alternative methods to bridge this gap. Ingber *et al*. developed a generic ELISA using an engineered immunoglobulin domain fused to domains of mannose-binding lectin (termed FcMBL) that binds lipoglycan targets, including LPS and LTA, from a wide range of Gram-negative and Gram-positive bacteria directly in whole blood in less than 1 hour^59^. Their assay displayed a sensitivity of >80% in patients diagnosed with sepsis but lacks the ability to distinguish specific pathogens^59^. This detection strategy could be adapted for use on our platform, which displays at least 10-fold greater sensitivity in detecting LPS and LTA. In addition to this, however, our methods can also distinguish Gram-negative from Gram-positive pathogens, which is a significant advantage for suitable therapeutic intervention.

The accurate, reliable and rapid detection of LPS and LTA in blood, specifically at the point-of-care has, however, remained elusive. Most methods for detection of LPS at the point of need actually are measures of endotoxin activity, which not all LPS display, and not direct measurement of the antigen itself^60^. We and others have extensively reviewed methods for the detection of LPS, both rapid and time-consuming^59–62^. To our knowledge, there are no point of care methods for the direct and sensitive measurement of LPS from blood, other than the one presented in this manuscript.

There are several sandwich immunoassays and imaging methods for the measurement of LTA in physiological matrices such as bone. To our knowledge, only one publication reports the detection of the antigen with monoclonal antibodies from blood^63^, albeit after the extraction of the antigen *via* a method called ISOLATOR. Aviva Systems Biology produces a commercial LTA measurement kit, with a sensitivity of 20 ng/mL in 3 hours. The kits are not tailored for measurement of lipoprotein-associated LTA, and also take greater time and effort to completion compared to the assay reported in this manuscript.

Many bacterial PAMPs, beyond LPS and LTA, are amphiphilic molecules, which our team and others have shown are transported within the body by host serum lipoproteins^28–30^. Understanding this interaction has allowed for the development of a suite of assays for the direct and sensitive detection of this elusive category of biomarkers in aqueous blood. The development of a quick sample processing method eliminates the need for prior knowledge of the interactions between bacterial PAMPs and host lipoproteins. Herein, we have applied this strategy towards the diagnosis of bacteremia and sepsis in children from a high-disease burden population. Our findings indicate that a sensitive and rapid approach, albeit one with lower specificity than methods targeting nucleic acids, is sensitive enough to facilitate timely intervention and, therefore, has the potential to reduce mortality. In fact, recent work from our group has already shown that a diagnostic tool with low specificity, but high sensitivity, significantly reduces mortality in high-disease burden area^54^. In the future, this approach can be evaluated for its value in guiding antibiotic use, as well as a prognostic indicator.

## Methods

### Reagents and Materials

PBS was purchased from Sigma-Aldrich (D8662). Bovine serum albumin (BSA, A7906) and Dulbecco’s phosphate buffered saline (PBS, D1408) were purchased from Sigma Aldrich (St. Louis, MO). Control human serum (normal pool) was purchased from Fisher BioReagents (BP2657100), 1 mL aliquots were stored at −20 °C and thawed only once prior to use. Goat serum was purchased from Thermo Fisher Scientific (16210064), donkey serum was purchased from EMD Millipore Corp (S30-100ML), and prior to use both were diluted 1:1 with an equal volume of PBS. Alexa Fluor 647 conjugated streptavidin (S21374) was obtained from Thermo Fisher Scientific. 1-Step Ultra TMB-ELISA Substrate Solution (34028) and Nunc MaxiSorp flat-bottom 96 well plates (44-2404-21) were purchased from Thermo Fisher Scientific.

### Antibodies

#### LPS

The Salmonella Polyclonal Antibody from Thermo Fisher Scientific, catalog number PA1-7244, RRID AB_561201 and was referred to as α-LPS.

#### LTA

The Lipoteichoic Acid Monoclonal Antibody (55) from Thermo Fisher Scientific, catalog number MA1-40134, RRID AB_1076514 and was referred to as α-LTA. The mouse monoclonal α-Gram Positive Bacteria antibody (called α-Gram+) was purchased from abcam (ab20344; Cambridge, MA). The rabbit polyclonal IgG α-Staphylococcus aureus antibody (called α-Sau pAb) was purchased from abcam (ab20920). LPS and LTA antibodies were fluorescently-conjugated using Alexa Fluor® 647 (AF647) antibody labeling kits purchased from Life Technologies (Thermo Fisher Scientific, Grand Island, NY) according to manufacturer instructions including determination of the antibody concentration and degree of labeling using a NanoDrop 1000 instrument (Thermo Fisher Scientific). AF647 yields an emission with maximum RFU at 665 nm. HDL/LDL. The goat polyclonal IgG α-apolipoprotein AI antibody and α-apolipoprotein B antibody conjugated to biotin were purchased from abcam (ab27630 and ab20898). Secondary antibodies. HRP-conjugated α-rabbit antibody was purchased from Thermo Fisher Scientific (31300) and α-mouse was purchased from Dako (D0486).

### LPS

Purified LPS from *Salmonella* Typhimurium (catalog no. 225) was obtained from List Biological Laboratories, Inc. (Campbell, CA) or Sigma-Aldrich (St. Louis, MO) for *Salmonella* Typhimurium (L6511), *Escherichia coli* O111:B4 (L2630), *Klebsiella pneumoniae* (L4268) and *Pseudomonas aeruginosa* (L9143). LPS was resuspended to 5 mg/mL in sterile nanopure water. 100 µL aliquots were stored in glass vials at −20 °C and thawed immediately prior to use. Additionally, LPS was purified in by chloroform-methanol extraction from *Salmonella* Typhimurium reference strain ATCC 14028, which was obtained from the American Type Culture Collection (ATCC, Manassas, VA). Total lipid extraction on bacterial cells was performed using a chloroform-methanol extraction method described by Bligh and Dyer^64^. Briefly, *S*. Typhimurium 14028 was streaked from a frozen stock onto Tryptic Soy Agar (Becton Dickinson) plates and incubated overnight at 37 °C. The resulting colonies were scraped in to a pre-weighed centrifuge tube, suspended in 10 mL of sterile water and pelleted at 4,000 × g for 20 min. The supernatant was discarded and for every 1 mL of pellet, 10 mL of a chloroform +10 mL of methanol +3 mL of water were added. The pellet was resuspended on a rocker shaking at room temperature for 2 hours. The sample was then centrifuged at 4,000 × g for 20 min to allow for phase separation. The organic supernatant containing the lipid lysate was transferred to a sterile glass tube. If cloudy, the extraction was repeated. The lipid lysates were stored at −80 °C until further use.

### LTA

Purified LTA from *Staphylococcus aureus* (L2515), *Streptococcus pyogenes* (L3140), *Bacillus subtilis* (L3265), and *Enterococcus hirae* (L4015) was purchased from Sigma-Aldrich (St. Louis, MO) and resuspended to 5 mg/mL in sterile nanopure water. 100 µL aliquots were stored in glass vials at −20 °C and thawed immediately prior to use.

### Waveguide-based Optical Biosensor

The waveguide-based optical biosensor was developed at the LANL and is described in detail elsewhere^41,46,47^. The planar silicon oxynitride (SiONx) optical waveguides were fabricated at nGimat Ltd (Atlanta, GA) and coated with a 10 nm surface of SiO_2_ to enable functionalization at Spectrum Thin Films Inc. (Hauppauge, NY). Silicone gaskets for waveguide assembly were purchased from Grace Bio-Labs (Bend, OR). Glass microscope slides used as coverslips were purchased from Thermo Fisher Scientific (Rockford, IL).

### Waveguide Preparation and Flow Cell Assembly

Single mode planar optical waveguides were cleaned and prepared as described previously^36,46,47,51^. Briefly, waveguides and cover slides were sonicated in a water bath for 5 min sequentially in chloroform, ethanol, and then nanopure water. Next, waveguides and cover slides were dried completely under argon gas and irradiated using UV-ozone (UVOCS Inc., Montgomeryville, PA) for 40 min and stored dry until use. Flow cells for immunoassays were assembled using clean waveguides and cover slips, which were bonded together with a silicone gasket containing a laser cut channel creating a flow cell. Following assembly, the flow cell was injected with 70 µl of lipid micelles (preparation described below) and then incubated overnight at 4 °C to facilitate vesicle fusion and lipid bilayer stabilization^46^.

### Lipid Micelle Preparation

1,2-Dioleoylsn-glycero-3-phosphocholine (DOPC) and 1,2-dioleoyl-sn-glycero-3-phosphoethanolamine-N-(cap biotinyl) (sodium salt) (cap biotinyl) were obtained from Avanti Polar Lipids (Alabaster, AL), resuspended in chloroform and stored at −20 °C. Lipid micelles for use in waveguide experiments were prepared as described previously^36,41,47^. Briefly, 2 mM DOPC and 1% cap biotinyl (mol/mol) were combined in a glass tube then the chloroform was evaporated off under argon gas. Lipids were rehydrated in PBS, incubated in the dark for 30 min at room temperature shaking (100 rpm) on an orbital shaker. Lipid solutions then underwent 10 rapid freeze/thaw cycles alternating between liquid nitrogen and room temperature water. Finally, lipids were probe sonicated for 6 min total (1.0 sec pulse on/off, 10% amplitude) using a Branson ultrasonic generator. Once the lipids are stabilized, the addition of biotin allows for the bilayer integrity to be evaluated during immunoassay experiments by probing with 50–100 pM of a streptavidin Alexa Fluor 647 conjugate^35,46^.

### Waveguide-based Assays

Two novel assay strategies were developed for the detection of amphiphilic PAMPs in patient samples. All incubations occurred at room temperature. Dilutions of all reagents were made in PBS. All assays were performed in duplicate (unless otherwise specified). Flow cells were prepared as described above and the lipid bilayer was blocked for 1–2 hr with 2% BSA in PBS (w/v). All incubations were immediately followed by a wash with 2 mL of 0.5% BSA in PBS (w/v) to remove any unbound constituents. Incident light from a 635 nm laser (Diode Laser, Coherent, Auburn, CA) with power adjusted to 440–443 µW was coupled into the waveguide using a diffraction grading. The response signal was adjusted for maximum peak intensity using a spectrometer (USB2000, Ocean Optics, Winter Park, FL) interfaced with the instrument and an optical power meter (Thor Labs, Newton, NJ)^36,40,41^. Resulting spectra from the waveguide biosensor were processed and displayed graphically using Igor Pro 6.37. Raw spectral curves were integrated between 550 and 850 nm using a 647 nm long pass filter in order to capture the signal of interest from Alexa Fluor® 647.

The background signal associated with the lipid bilayer and 2% BSA block was recorded, and then the integrity of the lipid bilayer is assessed by incubation of 50–100 pM streptavidin, AF647 conjugate (Molecular Probes, S32357) for 5 min. The remaining assay steps depend on the particular assay and antigen (LPS or LTA) tested as described below and depicted in Table S7. The incubation times for the selected antigens and detection antibody were optimized in all cases by standard measurements using LPS spiked into commercially procured human serum. The antigens and antibody titrations were performed on the waveguide-based biosensor.

#### Membrane Insertion Assay

The non-specific signal between the detection antibody (conjugated to Alexa Fluor® 647) and the lipid bilayer was then determined by incubation of detection antibody. Then LPS or LTA was incubated allowing for association with the lipid bilayer. Lastly, the detection antibody was incubated and the specific signal associated with the antibody and LPS or LTA associated with the lipid bilayer was measured. Raw data was recorded as relative fluorescence units (RFU) as a function of wavelength (nm).

#### Lipoprotein Capture Assay

The lipoprotein capture assay utilized capture antibodies directed against the coat proteins of HDL and LDL nanodiscs (α-apoA1 and α-apoB). This allows for the capture of LPS associated with HDL and LDL nanodiscs in serum. Following the test for lipid bilayer integrity, 10 nM unlabeled streptavidin was added and incubated for 10 min to saturate the biotin embedded in the lipid bilayer. Subsequently, 100 nM of biotin conjugated α-apoA1 (α-HDL) and/or α-apoB (α-LDL) antibody was incubated for 45 min. For assays capturing both HDL and LDL, 100 nM of each was combined and injected simultaneously. Then the non-specific signal was determined by incubation of the detection antibody followed by addition of LPS or LTA incubation allowing for association of the biomarker-lipoprotein complex with the capture antibody. Lastly, the specific signal was measured following the addition of the detection antibody. Raw data was recorded as RFU as a function of wavelength (nm). The specific/non-specific ratio (S/N) was determined by taking the maximum RFU value for the specific signal, subtracting out the maximum RFU value for the background and dividing this by the maximum RFU value for the non-specific signal minus the maximum RFU value for the background [Eq. (1)].

1 $$S/N=\frac{({Specific}-{Background})}{({NS}-{Background})}$$

#### LPS Assay

The α-Salmonella pAb antibody (PA1-7244) was used at a concentration of 25 nM as the LPS detection antibody with an incubation time of 90 min each for the non-specific and specific steps. Purified LPS was diluted to the desired concentration in either PBS or human serum in high-recovery glass vials (Thermo Scientific, C5000-995) and either used immediately or incubated overnight (18–24 hrs) at 4 °C to allow for association with lipoproteins in serum. The LPS sample was then injected into the flow cell and incubated for 2 h.

#### LTA Assay

The following were used as detection antibodies at a concentration of 25 nM with an incubation time of 30 min: α-Gram+ monoclonal antibody (ab20344) and α-Sau polyclonal antibody (ab20920). Also, a detection antibody cocktail containing 25 nM α-Gram+ and 25 nM α-Sau combined together was used when indicated. Purified LTA was diluted to the desired concentration in either PBS or human serum in high-recovery glass vials and either used immediately or incubated overnight (18–24 hrs) at 4 °C to allow for association with lipoproteins in serum. The LTA sample was then injected into the flow cell and incubated for 1 h.

### Sample Processing

The sample processing was performed using a modified single-phase Bligh and Dyer chloroform:methanol extraction^64^. Chloroform, methanol and the serum sample were combined in a siliconized microfuge tube (Fisher Scientific, 02-681-320) at a 1:2:0.8 (v/v) ratio. The mixtures was combined by gentle pipetting using low-retention pipet tips to avoid lipid adherence to the plastic, and then the mixture was centrifuged for 1 min at 2,000 × g to separate the proteins (supernatant) from the lipid/amphiphilic molecules (pellet). The supernatant was discarded and the biomarker-containing pellet was resuspended in PBS by gentle pipetting. Following a 5 sec pulse spin to settle debris that could clog the septum of our biosensor flow cell, the biomarker-containing solution was used as the biomarker sample for immunoassays. In the case of clinical samples, 40 µL of patient serum was combined with 100 µL of methanol and 50 µL of chloroform. Following processing, the biomarker-containing pellet was resuspended in 100 µL of PBS, pulse spun for 5 sec to remove debris, and then the remaining liquid (~80 µL) was used for detection assays.

### ELISAs

#### Cross-Reactivity

LPS and LTA were measured in ELISA format with all steps performed at room temperature. First, 96-well microtiter plates (Nunc Maxisorp Plates, Thermo Fisher, 442404) were coated with 100 µl of 25 µg/mL of the LPS or LTA diluted in PBS/0.02% Tween-20/0.5% bovine serum albumin and incubated for 30 minutes. After washing three times with PBS/0.5% Tween-20, the plates were then blocked with 200 µL PBS/0.02% Tween-20/0.5% bovine serum albumin for 30 min. The plates were washed three times with PBS/0.5% Tween-20 and 100 µL of the specific antibody was added and incubated for 1 hour. The polyclonal α-LPS antibody (Thermo Fisher Scientific, PA1-7244) was used at a 1:50 dilution of a 0.1 mg/mL stock in PBS LTA cocktail was comprised of a 1:50 dilution (0.1 mg/mL stocks) of both the α-Gram+ monoclonal antibody (Abcam, ab20344) and α-Sau polyclonal antibody (Abcam, ab20920). Following three washes, 100 µL of HRP-conjugated secondary antibody (α-rabbit for LPS and α-rabbit + α-mouse for LTA assay) was added to each well and incubated at room temperature for 30 min. The plates were washed four times (PBS/0.5% Tween-20) and 100 µL of 1-Step™ Ultra TMB-ELISA Substrate Solution (Thermo Scientific 34028) was added to each well. The reaction was stopped by adding 100 µL of H_2_SO_4_ (2 M) and absorbance measurements at 450 nm were taken using a Spectra Max VersaMax plate reader (Molecular Devices, Sunnyvale, CA). The average A_450_ for each condition was determined (n = 4). Error bars are ± standard deviation. Non-specific binding measurements were taken using PBS control wells in the absence of LPS or LTA.

#### Lipoprotein Capture

LPS and LTA were measured in ELISA format over varying concentration ranges. First, 96-well microtiter plates (Nunc Maxisorp Plates, Thermo Fisher, 442404) were coated with 100 µl of 50% serum diluted in PBS (goat serum for LPS, donkey serum for LTA) and incubated overnight at 4 °C. The serum contains lipoproteins that serve as capture molecules for LPS and LTA. The plates were then blocked with 300 µL PBS/0.02% Tween-20/0.5% bovine serum albumin for at least 1 h at room temperature. The plates were washed three times with PBS/0.5% Tween-20 before adding 100 µL of LPS or LTA diluted to the concentration indicated in blocking buffer, which was incubated for 2 h at room temperature. The plates were washed three times with PBS/0.5% Tween-20 and 100 µL of the specific antibody was added and incubated for 90 min at room temperature. The polyclonal α-Salmonella (Thermo Fisher Scientific, PA1-7244) was used at a 1:2000 dilution in PBS while the monoclonal α-lipoteichoic acid antibody (55) (Thermo Fisher Scientific, MA1-40134) was used at a 1:50 dilution in PBS. Following three washes, 100 µL of HRP-conjugated secondary antibody (α-rabbit for LPS and α-mouse for LTA assay) was added to each well and incubated at room temperature for 1 h. The plates were washed four times (PBS/0.5% Tween-20) and 100 µL of 1-Step™ Ultra TMB-ELISA Substrate Solution (Thermo Scientific 34028) was added to each well. The reaction was stopped by adding 100 µL of H_2_SO_4_ (2 M) and absorbance measurements at 450 nm were taken using a Spectra Max M2e plate reader (Molecular Devices, Sunnyvale, CA). The average A_450_ for each condition was determined (n = 3). Error bars are ± standard deviation. Non-specific binding measurements were taken using PBS control wells in the absence of LPS or LTA.

### Limit of Detection

The limit of detection (LOD) was obtained as described in Eq. (2). For a given sample concentration, the average non-specific signal for all replicates was obtained and added to three times the standard deviation (σ), multiplied by the sample concentration, and divided by the average specific signal for that concentration. Sample concentration and LOD will be in the same units, so if sample concentration is in ng/mL then LOD will be in ng/mL.

2 $${LOD}=\frac{(NS+3{\rm{\sigma }})[{\rm{Sample}}]}{{Specific}}$$

### Ethics

The scientific and ethical review committees of the Kenya Medical Research Institute and the institutional review boards of the University of New Mexico and LANL approved all protocols in this study involving human patients. All samples were collected following written informed consent by the parents or guardians of the patient, and patients participating in this study were treated according to the Kenyan Ministry of Health guidelines.

### Human Patient Samples

Clinical samples were obtained from pediatric patients (ages 4–25 months) recruited at the Siaya County Referral Hospital in western Kenya between 2004 to 2014 as part of a study examining the pathogenesis of severe malarial anemia and related co-morbidities. Blood samples were collected and tested for bacteremia as described previously^4^. Briefly, venous blood (~1 mL) was collected aseptically into sterile pediatric Isolator microbial tubes (Wampole Laboratories, Princeton, NJ) and inoculated directly onto chocolate agar plates. These plates were incubated for 18 h at 37 °C in 5% CO_2_ followed by 18–24 h subculture in an inverted position and inspected for bacterial growth. If no growth was observed after 24 h of subculture then plates were incubated for 4 additional days, and checked daily for bacterial growth. Following morphological observations, positive plate cultures were subject to Gram staining, biochemical testing (API biochemical galleries, bioMerieux, Louvres, France) and agglutination serology.

Venous blood (<3.0 mL) samples were obtained from the study participants prior to any treatment interventions. Thick and thin peripheral blood smears were prepared from venous blood samples and stained with Giemsa reagent for malaria parasite identification and quantification by microscopy. Asexual malaria parasites were counted against 300 leukocytes, and parasite densities were determined by multiplying the parasite count by the total leukocyte counts from an automated hematology analyzer (Beckman Coulter® AcT diff2™, Beckman-Coulter Corporation, Miami, USA).

Serum samples were isolated from venous blood and stored at −80 °C until use. Samples were thawed immediately prior to injection in the waveguide assays. If multiple assays were performed on a single serum sample, lipoprotein capture was performed first with the fewest freeze/thaw cycles to avoid degradation of lipoprotein carriers. For lipoprotein capture assays, 40–50 µL of clinical serum sample was diluted 1:1 with 40–50 µL of control human serum.

### Statistical Analysis

S/N ratios are presented as means ± standard deviation. Data were analyzed using one-way analysis of variance (ANOVA) with five degrees of freedom within groups. Fisher’s least significant difference (LSD) test was used for *post hoc* analysis to determine statistical significance between individual groups. A significance level (*P*) of less than 0.05 was considered statistically significant (***P < 0.001, **P < 0.01, or *P < 0.05).

## Supplementary information

Supplementary information

## Data Availability

Sequence data that support the findings of this study have been deposited in NCBI with the accession codes NHTP00000000, NHRB00000000, NHQY00000000, NHQX00000000, and NWTZ00000000. The raw spectral data that support the findings of this study are available from the corresponding author upon request.
