# Supplementary material for: Direct detection of bacteremia by exploiting host-pathogen interactions of lipoteichoic acid and lipopolysaccharide
Source: Sci Rep. 2019 Apr 17;9:6203. doi: 10.1038/s41598-019-42502-5 (PMC6470174; doi:10.1038/s41598-019-42502-5)
Supplement: Supplementary file 1 — Supplementary information [file 41598_2019_42502_MOESM1_ESM.docx]

**Direct detection of bacteremia by exploiting host-pathogen interactions of lipoteichoic acid and lipopolysaccharide**

Jessica Z. Kubicek-Sutherland, Dung M. Vu, Aneesa Noormohamed, Heather M. Mendez, Loreen R. Stromberg, Christine A. Pedersen, Astrid C. Hengartner, Katja E. Klosterman, Haley A. Bridgewater, Vincent Otieno, Qiuying Cheng, Samuel B. Anyona, Collins Ouma, Evans Raballah, Douglas J. Perkins, Benjamin H. McMahon, and Harshini Mukundan

**SUPPLEMENTARY TABLES**

**SUPPLEMENTARY FIGURES**

**Supplementary Figure 1. Cross reactivity of LPS and LTA antibodies.** The **(a)** α-LPS antibody and **(b)** cocktail of α-Gram+ monoclonal antibody (mAb) and α-Sau polyclonal antibody (pAb) cocktail (1:1) was used to detect LPS (black bars) and LTA (grey bars) from several bacterial strains, as compared to control (PBS only, white bars). All values given are the mean  ±  standard deviation. ELISAs were performed in quadruplicate (n = 4) and repeated at least twice. Statistical significance was determined by one-way ANOVA with Fisher's least significant difference test used for *post hoc* analysis (****P* < 0.001).
